# Management of Stenosing Tenosynovitis With the Eastwood Technique: Handling, Advantages, and Disadvantages

**DOI:** 10.7759/cureus.82702

**Published:** 2025-04-21

**Authors:** Guillermo Sergio Dorantes-Millan, Roberto Rodriguez-Ramirez

**Affiliations:** 1 Surgery, Hospital General Dr. Fernando Quiroz Gutierrez, Mexico City, MEX

**Keywords:** a1 pulley release, corticosteroid treatment, eastwood technique, hand surgery, minimally invasive surgery, outpatient surgery, stenosing tenosinovitis, surgical treatment, trigger finger

## Abstract

Stenosing tenosynovitis, or trigger finger, is a common condition resulting from repetitive use of the finger tendons, causing inflammation and narrowing of the flexor sheaths. This causes difficulty in sliding the tendon, which can lead to locking or pain when extending the fingers. The condition is associated with chronic diseases, such as diabetes and rheumatoid arthritis, and has a higher incidence in people between the ages of 40 and 60 years. Treatments include conservative approaches, such as the use of anti-inflammatory drugs and corticosteroids, or definitive approaches, such as surgery when previous treatments are unsuccessful. In this case, we present a 45-year-old man with type 2 diabetes mellitus who suffered from a trigger finger in the third finger of the right hand. After unsuccessful conservative treatment, he underwent A1 pulley release surgery using the Eastwood technique, resulting in uncomplicated improvement. This surgical approach has the advantage of being outpatient, minimally invasive, and offering a shorter recovery time. The success rate of this technique increases with the surgeon’s experience, being effective in most cases and allowing a quick return to daily activities.

## Introduction

Trigger finger or stenosing tenosynovitis is a prevalent condition arising from repetitive and mechanical use of the flexor sheaths of the fingers and thumb, resulting in a narrowing of the flexor sheaths [1,2], in addition to hypertrophy and inflammation at the tendon-sheath interface, leading to the widening of A1 pulley [3], which is anatomically located in the proximal section of the metacarpophalangeal joint [4,5].

The etiology of stenosing tenosynovitis is related to chronic degenerative diseases such as diabetes mellitus, rheumatoid arthritis, and amyloidosis [6,7]. Mechanical and traumatic forces are known to cause hypertrophy of the tendon and its sheath as a consequence of the inflammation generated by the trauma [3,8], which causes, depending on the degree of entrapment, sensations of blockage in the mobility of the finger due to the inability to slide smoothly within its sheath [9,10].

Stenosing tenosynovitis is a common cause of disability characterized by pain and blockage of finger extension [3]. It has an annual incidence of 28 cases per 100,000 population, with a prevalence of 2.6%, and peaking between the fifth and sixth decades of life [3]. Trigger finger has been associated with carpal tunnel syndrome, Dupuytren’s disease, and inflammatory arthritis [4,6]. Among the most affected fingers are the first and third fingers of the dominant hand [10]. This disorder, although initially not very limiting, becomes of interest when it inhibits the patient’s daily functioning [3,5].

## Case presentation

A 45-year-old male engaged in a white collar job with a five-year history of type 2 diabetes mellitus was referred from his first contact care unit to our plastic surgery service with the diagnosis of trigger finger in the third finger of the right hand with a one-year evolution. The patient was treated conservatively with steroid injections, with an unfavorable evolution. At the time of physical examination, the patient reported pain and an inability to the extension of the third finger, with a classification of Grade 3 according to the Green classification, right hand, and a feeling of a nodule in the third metacarpophalangeal joint on the palmar face.

**Figure 1:**
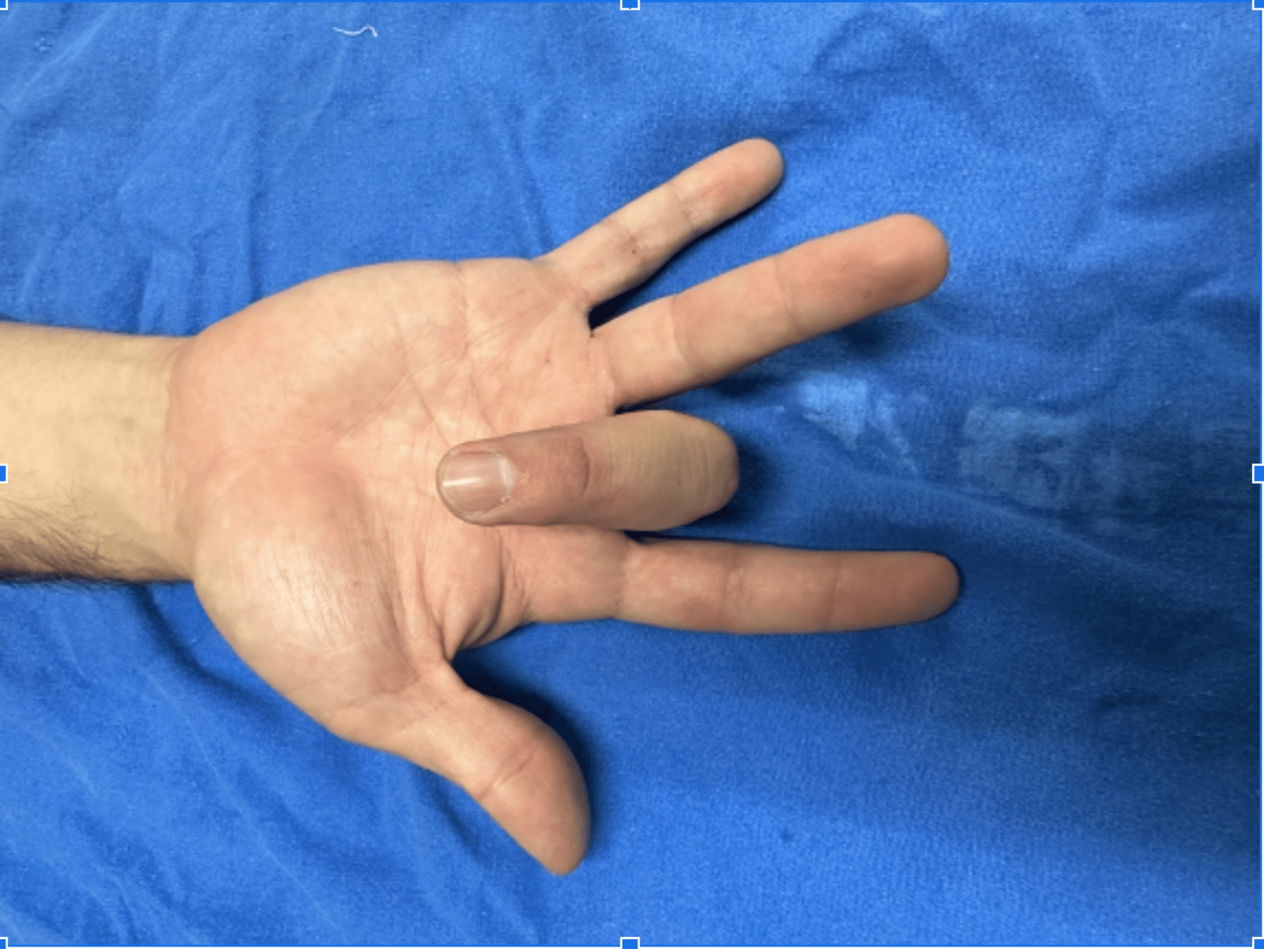
Inability to extend the third finger (Grade 3).

**Figure 2:**
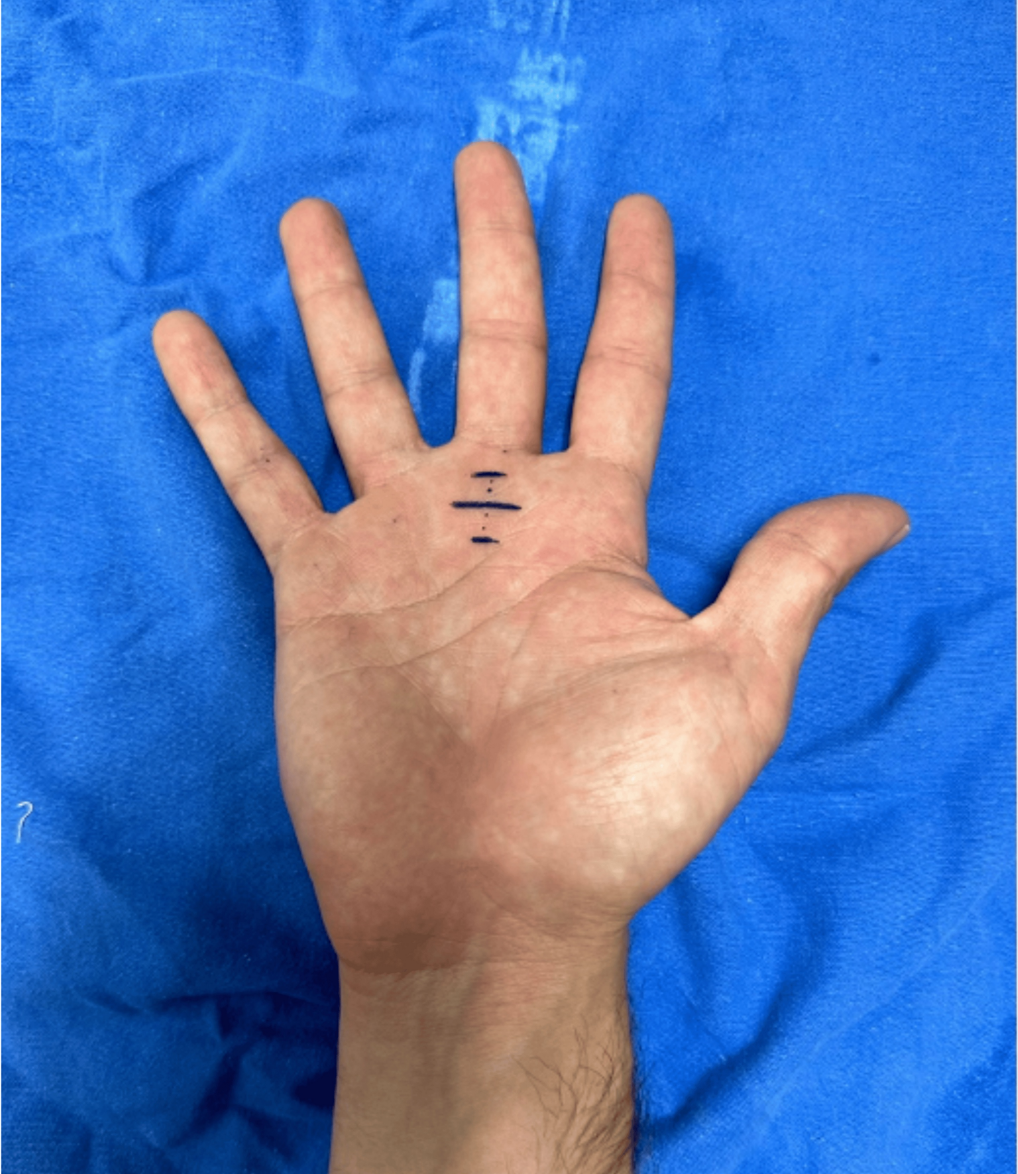
Hand in hyperextension.

**Figure 3:**
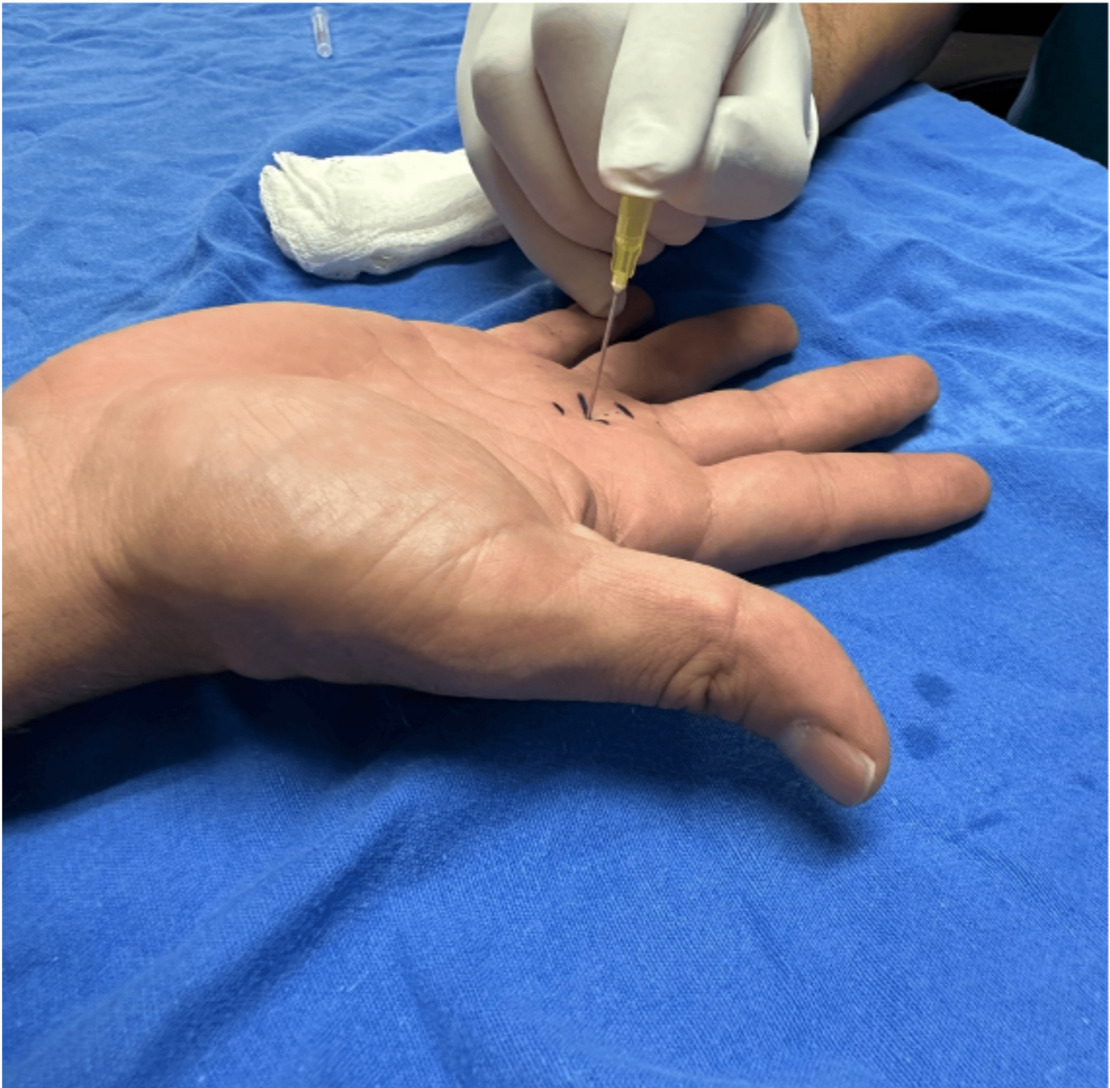
Hand in hyperextension.

**Figure 4:**
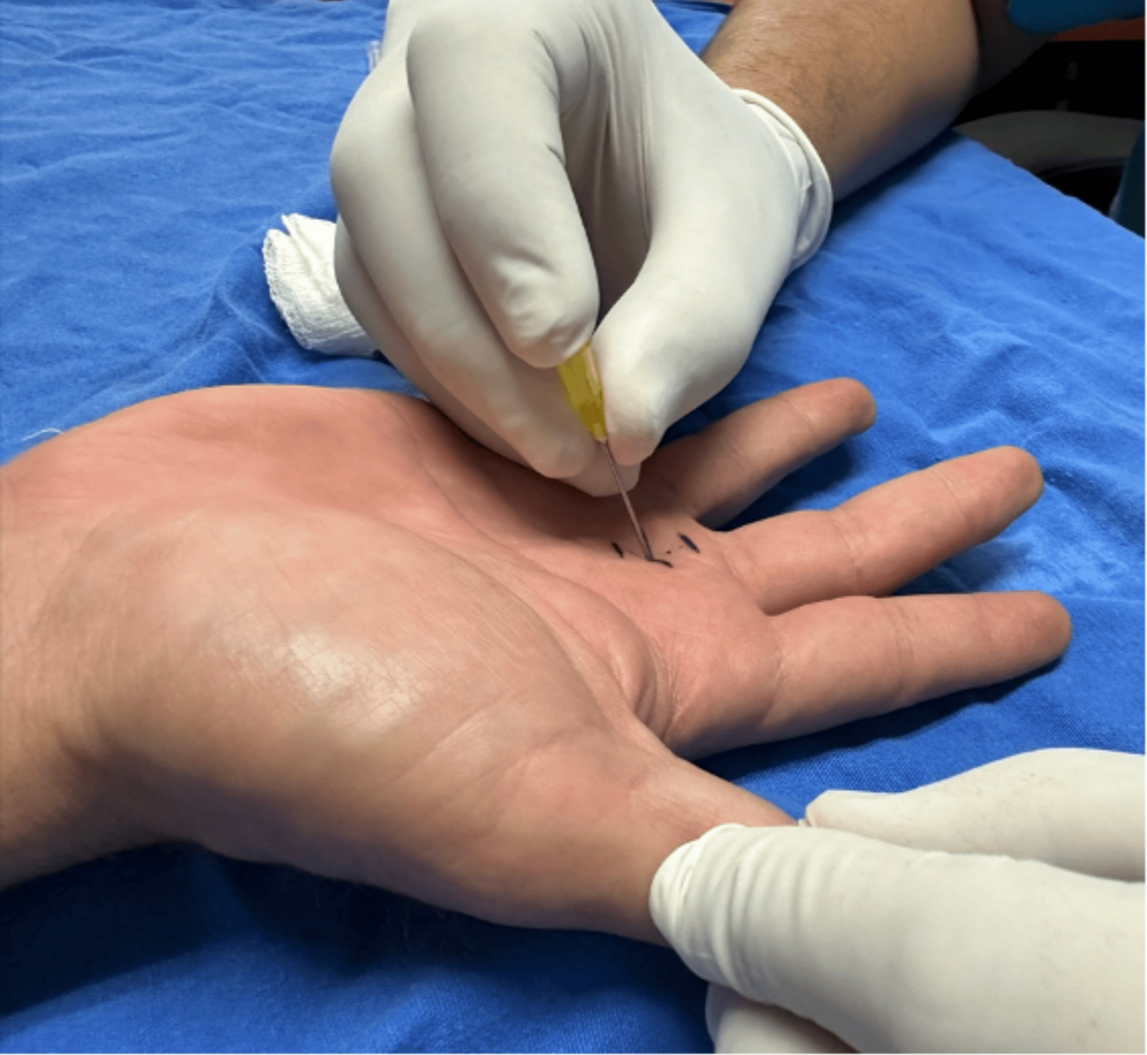
The Eastwood technique.

The patient reported hypersensitivity to palpation on the palmar side of the metacarpophalangeal joint of the third finger. When performing total flexion of the fingers of the right hand, there was an inability to perform extension of the middle finger, which required manipulation to extend the finger. A firm nodule was palpated in the region of the third metacarpophalangeal joint, and a diagnosis of Grade 3 stenosing tenosynovitis was made. A pre-surgical protocol was started, following which the patient was scheduled for outpatient surgery release. The surgical procedure was started after asepsis and antisepsis. The affected finger was placed in hyperextension (Figure 1). The area was infiltrated with 1% lidocaine at a rate of 2 mL in the subcutaneous tissue. At the level of the metacarpophalangeal joint, a 20-G needle was introduced into the flexor tendon, and 2 mm was withdrawn until the needle was resting on the A1 pulley. Once the release site was identified, the needle was moved in the longitudinal direction repeatedly until no resistance was perceived (Figure 2). Then, the patient was asked to perform flexion and extension of the finger to corroborate the release of the pulley. Subsequently, the needle was removed and the finger was flexed and extended to confirm that the blockage had disappeared (Figures 3, 4). Hemostasis was verified, and sterile gauze was placed. Finally, a semi-compressive bandage was applied to complete the surgical procedure. The patient was discharged on an outpatient basis, and an appointment was made for evaluation one week later at the outpatient clinic. The patient was evaluated one week after the surgery with favorable evolution, without recurrence, and limitation of hand movement.

## Discussion

The treatment of stenosing tenosynovitis includes both surgical and conservative approaches. The first one can act as definitive treatment, while the conservative approach can be definitive on rare occasions. As the first line of treatment, oral anti-inflammatory drugs are included. If these do not exert a favorable effect, treatment is continued based on the use of corticosteroids, which diminish the inflammation, resulting in a 50-90% reduction of the symptoms [1,3,6]. Due to the variability of the evolution, the impossibility to perform activities of daily life or work, and the lack of response to conservative treatment, surgical treatment is resorted to in most cases [8,9].

Once surgical treatment is decided, two techniques can be employed, namely, surgical treatment with open and percutaneous approaches [5,10]. The open surgical release of the A1 pulley has a success rate of 83-98% [4]. However, the main complications of this procedure are a prolonged recovery time, wound dehiscence, infection, and stiffness, unlike the percutaneous approach which has more advantages, including a shorter recovery time, avoiding the complications of an open surgical wound, and a success rate of over 91% with an almost immediate recovery of activities of daily living [5,8,9]. However, there is a potential risk of injury to the tendon, neurovascular structures, or failure in complete release, which is also seen in open releases [10].

## Conclusions

We present the case of a patient undergoing A1 pulley release surgery with the Eastwood technique, which is fast, effective, and avoids the complications of undergoing release by the open technique. The Eastwood technique has certain advantages, such as ambulatory, minimally invasive, and no morbidity, as well as disadvantages, such as anatomical variability, inexperience of the surgeon, and no visibility of the structures. The success rate of this surgery has been seen to be proportional to the surgeon’s experience, which is known to increase up to 98% after at least three years of experience. This accessible procedure can help avoid anesthetic complications in the operating room, as it is ambulatory. At the end of the release, patients can return to their daily lives and resume their activities almost immediately.

## References

[1] Matthews A, Smith K, Read L, Nicholas J, Schmidt E (2019). Trigger finger: an overview of the treatment options. JAAPA.

[2] Belloti JC, Sato ES, Faloppa F (2022). Trigger finger treatment. Rev Bras Ortop (Sao Paulo).

[3] Wu RT, Walker ME, Peck CJ (2023). Differential pulley release in trigger finger: a prospective, randomized clinical trial. Hand (N Y).

[4] Lee SH, Choi YC, Kang HJ (2018). Comparative study of ultrasonography-guided percutaneous A1 pulley release versus blinded percutaneous A1 pulley release. J Orthop Surg (Hong Kong).

[5] Merry SP, O'Grady JS, Boswell CL (2020). Trigger finger? Just shoot!. J Prim Care Community Health.

[6] Patrinely JR Jr, Johnson SP, Drolet BC (2021). Trigger finger corticosteroid injection with and without local anesthetic: a randomized, double-blind controlled trial. Hand (N Y).

[7] Johnson E, Stelzer J, Romero AB, Werntz JR (2021). Recognizing and treating trigger finger. J Fam Pract.

[8] Eastwood DM, Gupta KJ, Johnson DP (1992). Percutaneous release of the trigger finger: an office procedure. J Hand Surg Am.

[9] Fiorini HJ, Tamaoki MJ, Lenza M, Gomes Dos Santos JB, Faloppa F, Belloti JC (2018). Surgery for trigger finger. Cochrane Database Syst Rev.

[10] Çimen O, Nami Ş (2023). Does surgical experience affect the outcomes during percutaneous release of the trigger finger?. Cureus.

